# Effect of GFRP Stirrup Confinement on the Bond Strength of GFRP-RC Beams

**DOI:** 10.1186/s40069-024-00729-1

**Published:** 2024-12-12

**Authors:** Zahid Hussain, Jesús D. Ortiz, Seyed Arman Hosseini, Brahim Benmokrane, Antonio Nanni

**Affiliations:** 1 Civil and Architectural Engineering Department, University of Miami, Coral Gables, FL 33146 USA; 2 Department of Civil and Building Engineering, University of Sherbrooke, Sherbrooke, QC J1K2R1 Canada

**Keywords:** GFRP rebars, GFRP stirrups, Concrete, Bond strength, Development length, Confinement effect, Design codes

## Abstract

The current provisions for development length in the ACI 440.11 code disregard the confinement effect provided by stirrups on the bond strength of longitudinal bars and require splice lengths that pose implementation challenges. Given the significant improvement in GFRP material properties, this study investigated the bond strength of sand-coated GFRP bars and proposed a new factor to include the effect of stirrup confinement on the bond-strength provisions. The experimental program involved 16 GFRP-reinforced concrete (RC) beams having a width of 300 mm, and depth 440 mm, consisting of two repetitions for every configuration, subjected to four-point loading. The test parameters comprised lap-splice length and stirrup spacing in the lap-spliced zone. Out of 16 GFRP-RC beams, two beams were reinforced with two M16 (No. 5) continuous bars and six with varying lap-splice lengths [i.e., 40, 60, and 80 bar diameters (d_b_)] without confining stirrups. To evaluate the effect of confining stirrups, eight beams were reinforced with two M16 (No. 5) lap-spliced longitudinal bars (i.e., 40 and 60 d_b_) and M13 (No. 4) stirrups spaced at 100 mm (4 in.) and 200 mm (8 in.) center-to-center. Based on experimental results, stirrup confinement clearly increased the bond strength, reduced longitudinal bar slippage, and increased splitting stress. The beams with a splice length of 60 d_b_ and stirrups on 100 mm (4 in.) centers achieved 57% higher capacity than those with the same lap-splice length but without stirrups. Further, the ACI 440.11 equation overestimated the bond strength of sand-coated GFRP bars but yielded conservative results with closely spaced stirrups. CSA S6:25 predicted bond-strength values that were close to the experimental results compared to CSA S6:19, and CSA S806:12.

## Introduction

The corrosion vulnerability of steel bars adversely affects the durability of steel-reinforced concrete (RC) structures. Therefore, the use of steel bars should be reconsidered in favor of composite reinforcement, especially when structures are exposed to harsh environments. Currently, Glass FRP (GFRP) bars are the only composite reinforcement type allowed in the new ACI 440.11 code (ACI Committee 440, 2022). Despite facing challenges, construction projects worldwide have embraced the use of GFRP as primary or auxiliary reinforcement (Ortiz et al., 2023). GFRP's high tensile strength [up to 1,400 MPa (204 ksi)] and light weight (about one-fourth that of steel) provide construction advantages. Nonetheless, adequate bond strength between the rebar and surrounding concrete is necessary to attain the desired high tensile capacity. The bond strength of GFRP bars to concrete depends on several factors, including bar surface properties, bar diameter, concrete cover, embedded length, and amount of transverse reinforcement (Al-Salloum et al., 2022). There has been preliminary research on the bond performance of GFRP bars but, given the variety in surface treatments (i.e., sand-coated, spiral-wrapped, ribbed, and indented) and the properties of GFRP bars, the available data contains contradictions and has low reliability.

The current development-length equation in ACI 440.11 (2022) is based on the work of Wambeke and Shield (2006) and disregards the presence of stirrups. Disregarding stirrup confinement results in longer embedment lengths and might increase congestion in the lap zone and anchorage challenges at member terminations (Hussain & Nanni, 2023).

From the initial inclusion of the current development length equation in the ACI guideline pre-dating the Building Code, various research projects were dedicated to assessing and refining its accuracy. Aly et al. (2006) investigated the effects of varying bar diameters and splice lengths on the bond strength of FRP bars (GFRP and carbon FRP). They reported that the maximum stress of spliced FRP bars was linearly proportional to the splice length. Mosley et al. (2008) later reported it to be nonlinear and that the bond strength of GFRP bars depended on their elastic modulus. Pay et al. (2014) had similar findings, specifically that the bond strength of GFRP bars depended on axial rigidity. Esfahani et al. (2013) performed flexure and pullout tests on GFRP rebars embedded in concrete. The bond strength was reported to increase by 15–30% for ribbed bars in the presence of steel stirrups, whereas no considerable increase was observed for transversely confined sand-coated GFRP bars. The increase in bond strength for confined ribbed bars was attributed to their rib area. The authors reported that, for small values of transverse reinforcement or splices without transverse reinforcement, the ACI expression for development length was unconservative. Abbas et al. (2023) tested eight reinforced concrete beams reinforced with ribbed GFRP bars to investigate the effects of decreasing the embedded length and applying confining reinforcement. They found that lap splices designed according to the ACI 440.11 code (2022) were conservative in the presence of stirrups. In fact, the presence of steel stirrups and the spacing of the longitudinal GFRP rebars in the lap zone increased the bond strength by up to 31%. The increase in bond strength in the presence of steel stirrups has also been reported elsewhere using ribbed GFRP bars (Al-Salloum et al., 2022). Wu et al. (2022) tested 17 concrete beams reinforced with lap-spliced ribbed GFRP bars to investigate their bond behavior. The diameter of the GFRP rebars and the steel stirrup spacing were varied along the lap splice. The bond strength increased with the presence of steel stirrups but decreased as the stirrup pitch and bar diameter increased. Research studies investigating confined ribbed GFRP bars or conventional steel bars have concluded that bond strength depends on the relative rib area (Darwin et al., 1996; Esfahani et al., 2013; Wambeke & Shield, 2006). Accordingly, specimens with ribbed GFRP bars had better bond performance than sand-coated GFRP bars in the experimental tests by Esfahani et al. (2013).

Any increase in the confinement of the bar by surrounding concrete, transverse reinforcement, or bearing reaction increases the bond strength and minimizes the splitting forces (Tepfers, 1982). Hence, it is important to investigate all factors influencing the bond strength of GFRP bars to minimize the required development length and to enhance the overall reliability of the design provisions. Clearly, the abovementioned literature (summarized in Table 1) indicates that bond strength is positively affected by the confinement of the transverse reinforcement. Though researchers agree that bond strength increases in the presence of confining stirrups, such studies mainly investigated one type of longitudinal GFRP bars (i.e., ribbed) and used steel stirrups. Esfahani et al. (2013) provided some insights into the inferior performance of transversely confined sand-coated GFRP bars. The sand-coated bars used in that study had a modulus of elasticity of 37 GPa (5366 ksi). Since then, a new generation of high elastic modulus GFRP bars [i.e., 60 GPa (8700 ksi)] with better surface treatments has become available on the market. Consequently, the bond performance of sand-coated bars and the contribution of GFRP stirrups to bond strength need to be investigated again. This study was conducted to investigate the bond performance of sand-coated GFRP bars with different splice lengths and with or without the presence of confining GFRP stirrups in alignment with the overall scope of ACI 440.11, which currently does not cover the use of hybrid reinforcement. The results will help CODE committee to update the development length equation to include a factor signifying the impact of stirrup confinement on the bond strength.

**Table 1:** Bond strength of transversely confined GFRP bars

Authors	FRP type	Surface deformation	No. of specimens	Variables	Elastic modulus (GPa)
Mosley et al. (2008)	Glass	Indented, spiral wrap, braided	9	Reinforcement type, development length, and bar spacing	39.0
	Aramid				47.1
Esfahani et al. (2013)	Glass	Sand coated	3	Transverse reinforcement, surface properties, bar diameter	37.0
		Ribbed	10		60.0
Abbas et al. (2023)	Glass	Ribbed	8	Transverse reinforcement, stirrup spacing, rebar gap	52.0
Al-Salloum et al. (2022)	Glass	Ribbed	8	Transverse reinforcement, stirrup spacing, rebar gap	52.0
Wu et al. (2022)	Glass	Ribbed	22	Transverse reinforcement, stirrup spacing, bar diameter, loading	51.1
					48.8

## Methodology

### Material Properties

The main longitudinal reinforcement consisted of M16 (No. 5) GFRP bars with a nominal diameter of 15.9 mm (0.625 in.) and a nominal area of 200 mm^2^ (0.31 in^2^) as per ASTM D8505 (ASTM Committee D30, 2023). Stirrups were M13 (No. 4) GFRP bars with nominal diameter 12.7 mm (0.5 in.) and nominal area 129 mm^2^ (0.2). Table 2 presents the properties of the reinforcing bars of representative samples provided by the manufacturer.

**Table 2:** Properties of GFRP and steel rebars

Bar type	Nominal diameter (mm)	Cross-sectional area (mm^2^)	Yield strength (MPa)	Ultimate tensile strength (MPa)		Modulus of elasticity (GPa)	Strain (%)
				Average	Std. deviation		
GFRP	15.9	200.0	–	1326.0	30.1	64.9	0.020
	12.7	129.0	–	1126.0	16.8	52.4	0.021
	10.0	71.0	–	1614.0	37.7	65.9	0.024
Steel	15.9	200.0	415.0	–		200.0	0.002

All specimens were cast with standard structural ready-mixed concrete designed for a targeted compressive strength of 35 MPa (5070 psi). The concrete mixture incorporated a coarse aggregate with a nominal size of 19 mm (0.75 in.) and Type-I Portland cement; the slump was approximately 150 mm (6.0 in.). Three concrete cylinders of standard size—100 × 200 mm (4 × 8 in.)—for concrete compressive strength and two for splitting tensile strength were prepared from each concrete batch at the time of beam casting. Both beams and cylinders were demolded one day after casting and then moist cured for seven days under controlled laboratory conditions. The cylinders were tested according to ASTM C39 (ASTM Committee C09, 2021), showing an average compressive strength of 41.7 MPa (6048 psi) and a tensile strength equal to 4.3 MPa (625 psi). Table 3 provides the concrete compressive strength of individual beams, which was used for analysis. The concrete’s modulus of elasticity was computed according to ACI 440.11, Sect. 19.2.2.1(b), based on the 28-day compressive strength of the concrete; ${E}_{c}=4700\sqrt{{f{\prime}}_{c}}$ (SI).

**Table 3:** Beam design details

Beam ID	*f’*_*c*_ (MPa)	Splice length (*l*_d_) (mm)	*A*_tr_ (mm^2^)	Stirrup spacing (mm)	Cracking moment (*M*_cr_) (kN-m)	Expected moment (*M*_th_) (kN-m)	Design moment (*ɸM*_n_) (kN-m)	*M*_th_*/ɸM*_n_	*l*_d_*/d*_b_
G-C-00-1	36.0	Unspliced	–	–	30.5	153	100	1.53	-
G-C-00-2	36.0	Unspliced	–	–	30.5	153	100	1.53	-
G-40-00-1	42.9	630	–	–	33.3	163	106	1.63	39.8
G-40-00-2	40.7	630	–	–	32.3	160	104	1.54	39.8
G-60-00-1	42.9	950	–	–	33.3	163	106	1.53	60.1
G-60-00-2	40.7	950	–	–	32.3	160	104	1.54	60.1
G-80-00-1	42.9	1250	–	–	33.3	163	106	1.56	79.1
G-80-00-2	40.7	1250	–	–	32.3	160	104	1.53	79.1
G-40-100-1	44.4	630	129	100	33.8	165	107	1.54	39.8
G-40-100-2	42.0	630	129	100	32.8	162	105	1.54	39.8
G-40-200-1	44.4	630	129	200	33.8	165	107	1.54	39.8
G-40-200-2	42.0	630	129	200	32.8	162	105	1.54	39.8
G-60-100-1	44.4	950	129	100	33.8	165	107	1.54	60.1
G-60-100-2	42.0	950	129	100	32.8	162	105	1.54	60.1
G-60-200-1	43.3	950	129	200	33.4	164	107	1.53	60.1
G-60-200-2	43.3	950	129	200	33.4	165	107	1.54	60.1

*l*_*d*_  development length*, A*_tr_ stirrup area

### Test Specimens

This study comprised 16 GFRP-RC beams having a width of 300 mm (12 in.), depth of 440 mm (17.3 in.), and length of 5,200 mm (205 in.). The beam clear span was equal to 4800 mm (189 in.). The two reference GFRP-RC beams were reinforced with two continuous M16 (No.-5) GFRP bars in tension. Six GFRP-RC beams were reinforced with two M16 (No.-5) bars in tension with lap-splice lengths equal to 40, 60, and 80 d_b_ without stirrups in the spliced region. These lengths were selected to check the conservatism of the ACI- 440.11 development-length equation, which would require 102 d_b_. M16 (No. 5) steel rebars were used to hold the shear stirrups in place, but they were discontinued at the points of load application. Eight beams were reinforced with two M16 (No. 5) GFRP bars in tension with lap-splice lengths equal to 40, and 60 d_b_ and GFRP stirrups at 100 mm (4 in.) or 200 mm (8 in.) centers in the constant moment region. In this instance, two M10 (No.3) GFRP rebars were employed in the compression region throughout the length of the beam to hold the cage. Table 3 provides additional details about the beams. Two repetitions were implemented for each configuration to ascertain the quality of the results. The beams were notched at the end of the splice length to force cracking at the splice location. The notch width was equal to the width of the saw blade, the length equal to the width of the beam, and the depth 25 mm (1 in.).

A four-part notation was used for beam designation. For example, G-40-100-1 describes a GFRP-RC beam reinforced with two M16 bars (No. 5), 40 d_b_ splice length, a stirrup pitch of 100 mm (4 in.); the numeral 1 indicates the first repetition. All beams had a concrete clear cover equal to 38 mm (1.5 in.), which represents the minimum clear cover required for beams in accordance with ACI 440.11 (2022).

### Theoretical Capacity

Based on the provided data, expected cracking moment, design moment, and expected ultimate moment were computed in accordance with ACI 440.11 provisions (ACI Committee 440, 2022). The design moment (*ɸM*_n_) was calculated using the environmental reduction factor (*C*_*E*_), strength reduction factor (*ɸ*), and the rebar’s guaranteed tensile strength (*f*_fu_^*^). The *C*_*E*_ was taken equal to 0.85, and *f*_fu_^*^ was calculated as the ultimate tensile strength (*f*_u_) minus three standard deviations (*σ*), as required in ACI 440.11, Sect. 20.2.2.3. The specimen design was compression-controlled with two M16 (No. 5) bars. Therefore, the strength reduction factor (*ɸ*) was taken equal to 0.65 to calculate the theoretical capacity. Design capacities were calculated as provided in Table 3 using the specimen dimensions and reinforcement data. Reduction factors were not taken into consideration when determining the ultimate expected moment. The ratio of expected to design moment capacity highlights the inherent conservatism in ACI 440.11. It should be noted that self-weight of beam was considered during analysis of beams.

### Development Length as per ACI 440.11

The required development length (*l*_d_) was calculated for a M16 (No. 5) GFRP rebar based on ACI 440.11, Sect. 25.4.2, as given below:

1 $$l_{d} = \frac{{\left( {\frac{{f_{fr} }}{{0.083\sqrt {f^{\prime}_{c} } }} - 340} \right)\Psi_{t} }}{{13.6 + \frac{{C_{b} }}{{d_{b} }}}}d_{b} \left( {{\text{SI}}} \right)$$

where

- *f*_fr_ is the stress in the bar required to develop the full nominal sectional capacity (MPa). It is equal to *f*_fu_ for tension-controlled designs and the actual stress in the rebar for compression-controlled sections.
- *f*_fu_ is the design tensile strength of the GFRP longitudinal reinforcement (MPa), equal to the environmental factor (*C*_*E*_) times the guaranteed tensile strength (*f*_fu_^*^).
- *C*_*b*_ is the lesser of the distance from the center of a bar to the nearest concrete surface, or one-half the center-to-center spacing of the bars being developed (mm). The ratio between *C*_*b*_ and *d*_*b*_ shall not be greater than 3.5.
- *Ψ*_*t*_ is the bar location modification factor. It shall be 1.5 if more than 300 mm (12 in.) of fresh concrete is placed below the horizontal reinforcement being developed and 1.0 for all other cases.
- *f*_*u*_ is the mean tensile strength of a sample of test specimens (MPa).

The development-length value required to reach the full tensile capacity of the rebar (*f*_u_) calculated as per ACI 440.11 was equal to 102 d_b_. The lap-splice lengths used in this study were 40 d_b_, 60 d_b_, and 80 d_b_ or 39%, 60%, and 79% of the development-length value required by ACI 440.11. Note that, in this study, splice length is 1.0 × *l*_*d*_ where *l*_*d*_ is the development length calculated as per ACI 440.11 as shown in Eq. 1.

### Instrumentation

**Fig. 1:**
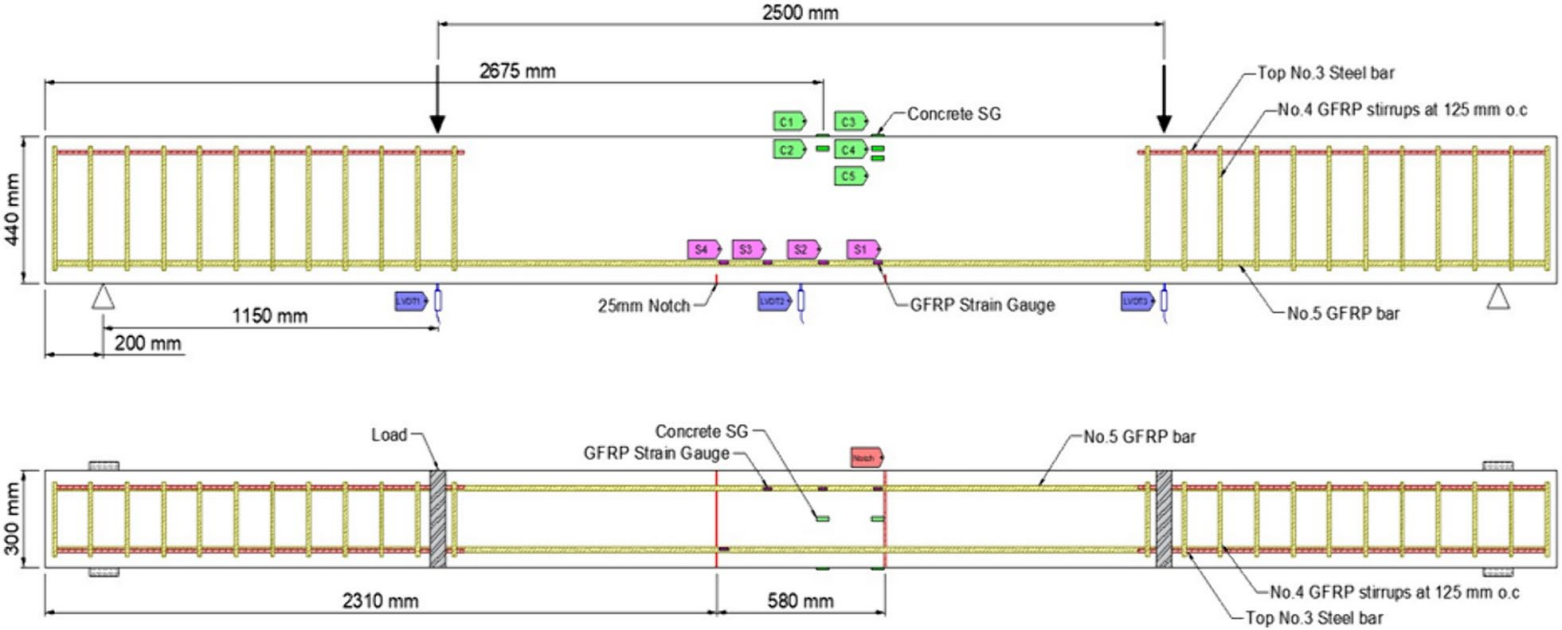
Continuous GFRP bars without confining stirrups in the splice region

**Fig. 2:**
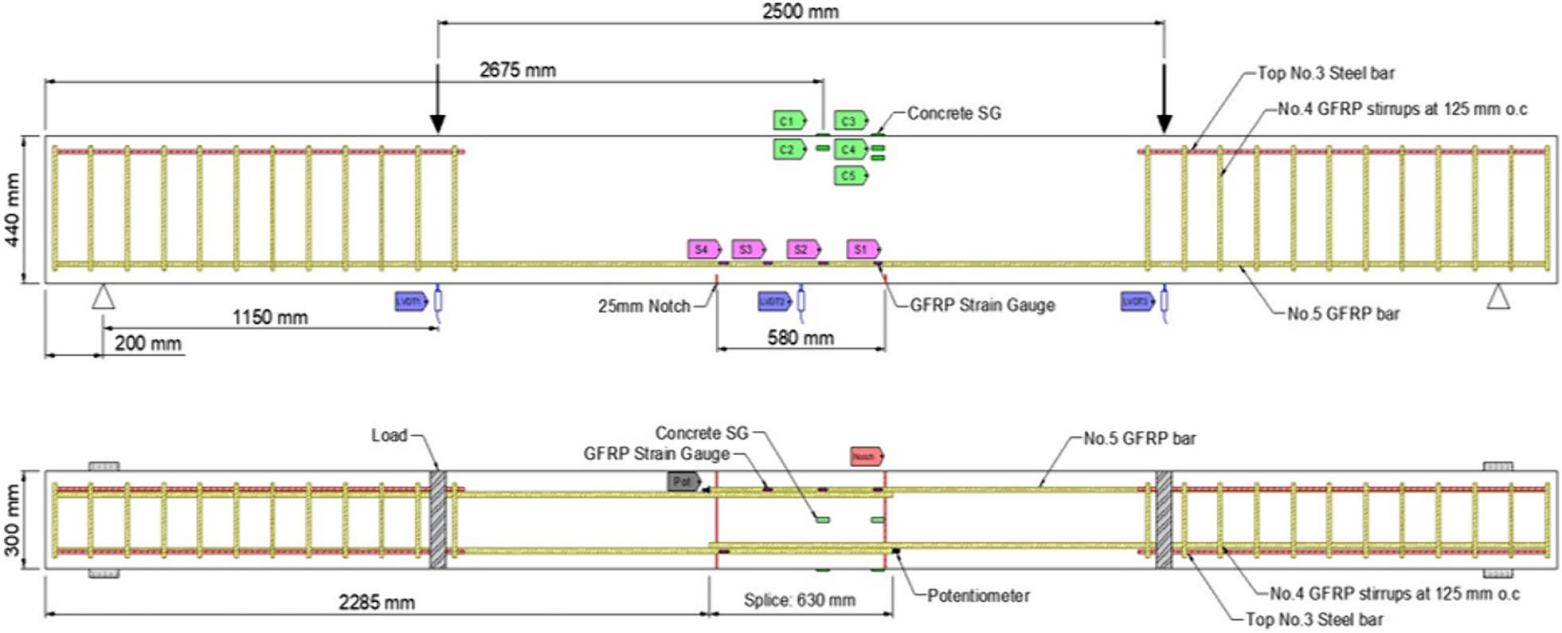
Spliced GFRP bars without confining stirrups in the splice region (40 d_b_ splice length)

**Fig. 3:**
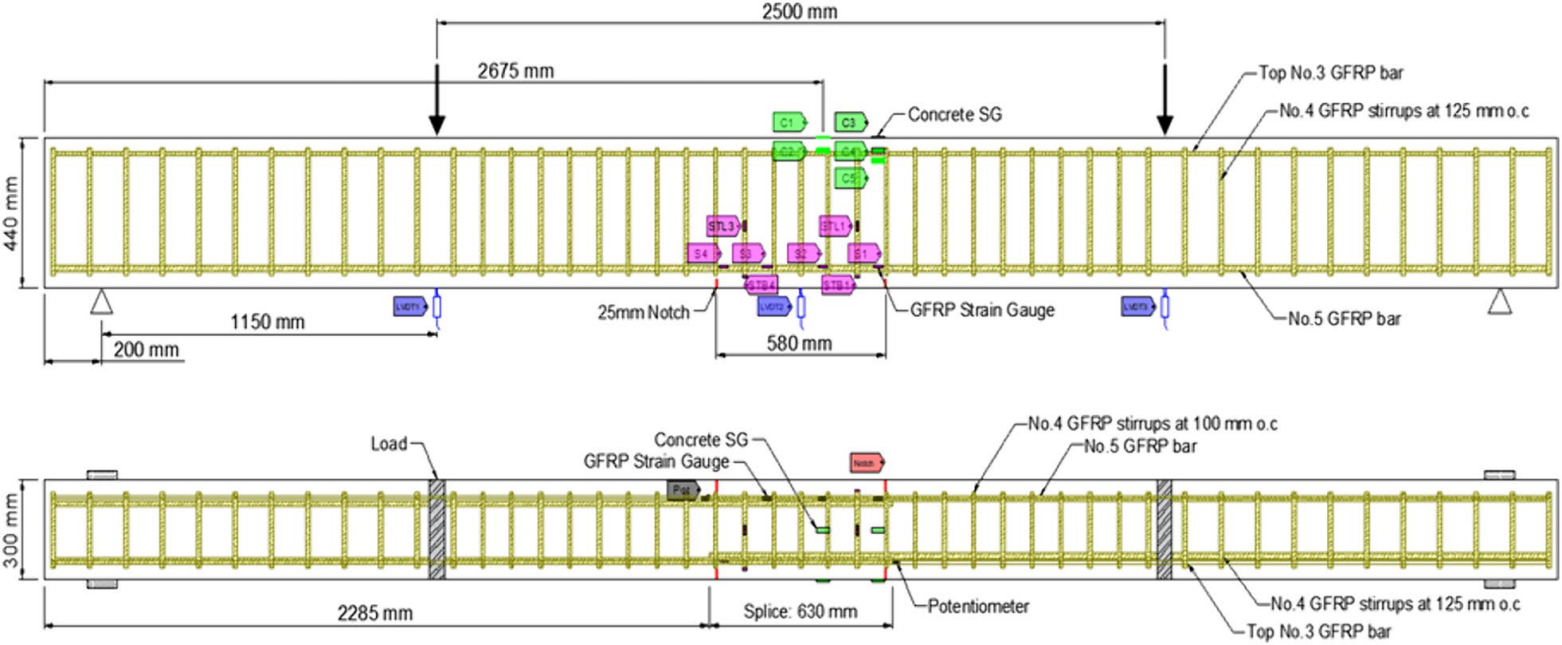
Spliced GFRP bars with stirrups in the spliced region (40 d_b_ splice length)

**Fig. 4:**
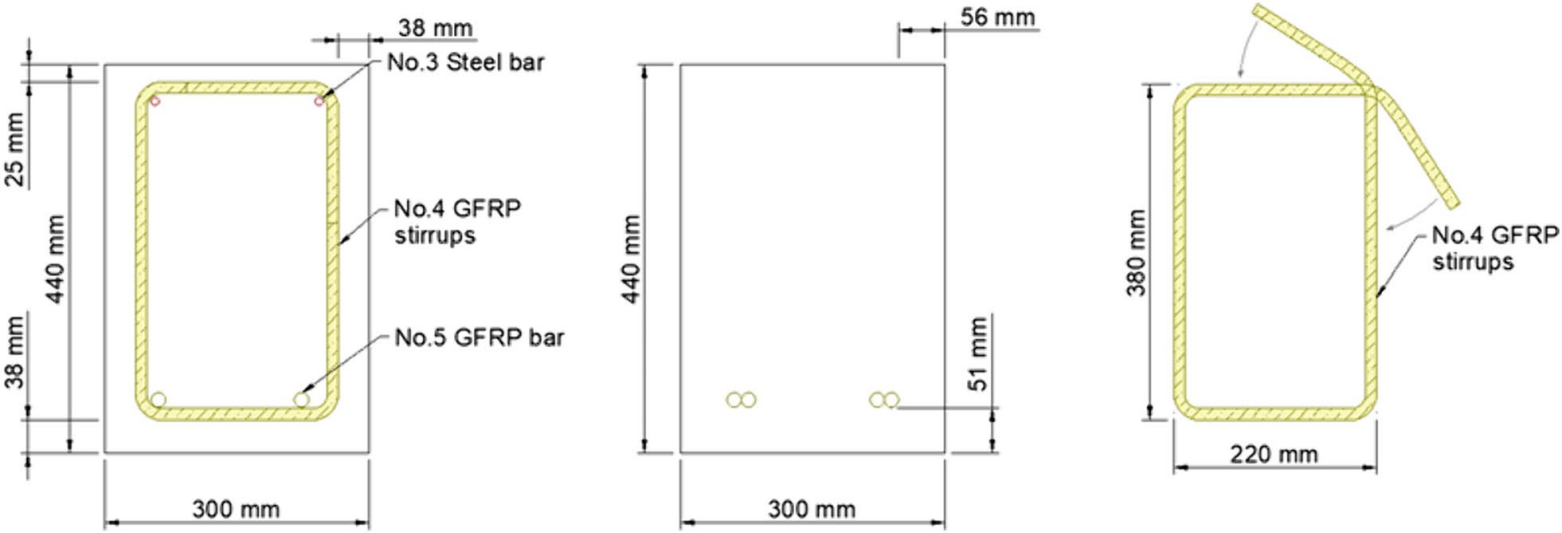
Cross-sectional view and stirrup details

**Fig. 5:**
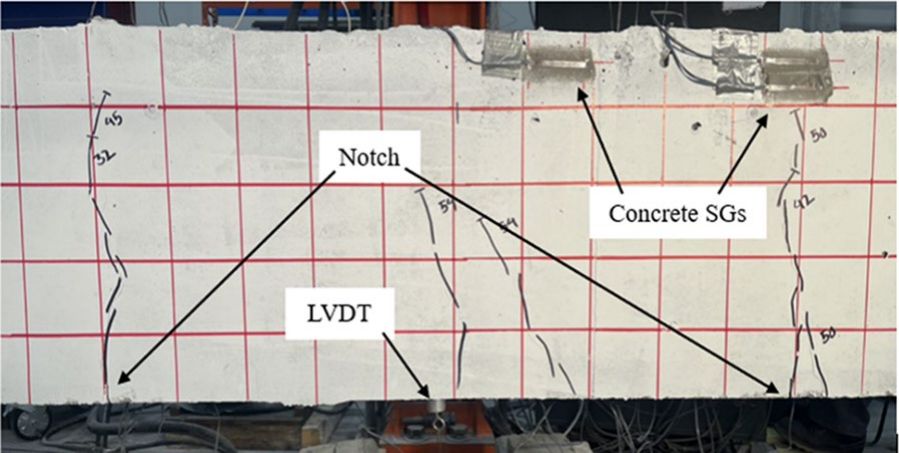
Concrete SGs and notch location

**Fig. 6:**
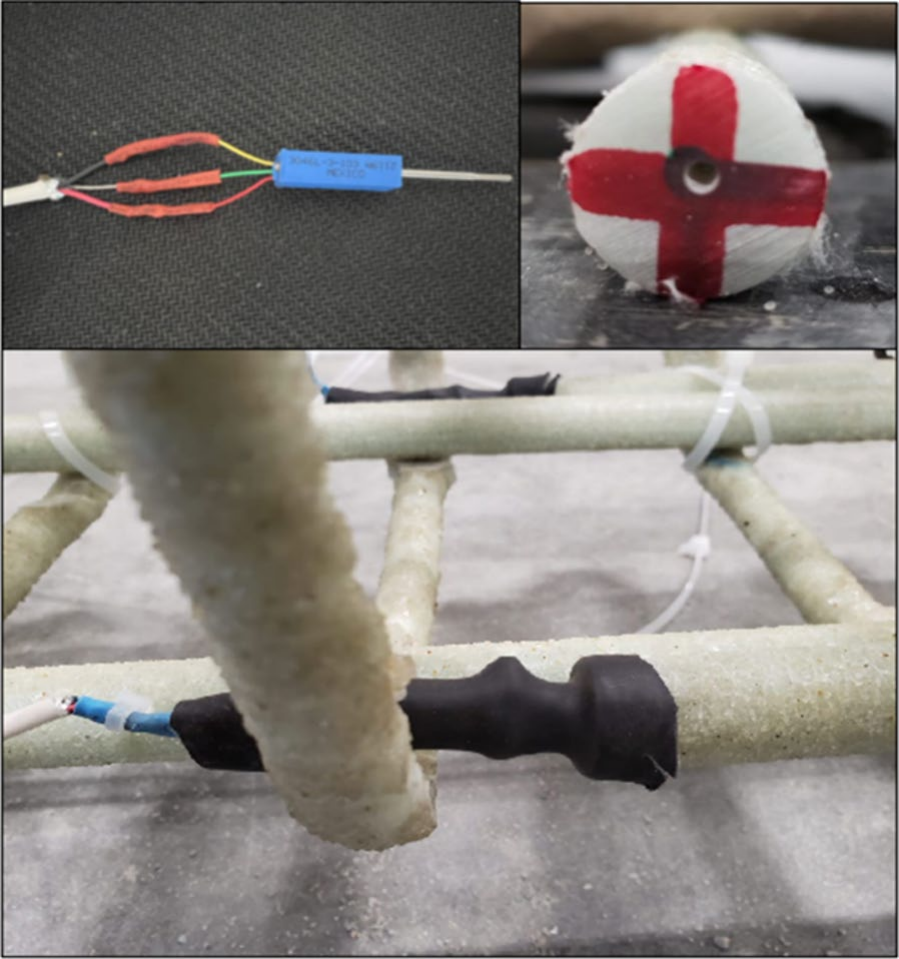
Potentiometer installation

Figs. 1, 2, 3, 4, 5, 6 provide the instrumentation details, including the measurement of vertical displacements using linear variable displacement transducers (LVDTs) and the strain in longitudinal bars, stirrups, and concrete with strain gages (SGs). Additionally, all beams with spliced bars were instrumented with potentiometers to measure the relative rebar slip. The potentiometers were installed by making a hole in the bar (See Fig. 6) and were protected with a heat-shrink tube to avoid damage during casting. The shaft of potentiometer was glued in the hole and space left for its free moment during testing. Three LVDTs were installed to monitor deflections: two under the loading knives and one at mid-span, as shown in Figs. 2 and 5. In the spliced beams, SGs with gage lengths equal to 6 mm (0.24 in.), gage factor 2.08 ± 1%, gage resistance 120 ± 0.5 Ω, and transverse resistance 0.4% were used. Three SGs were installed on the rebar on one side of the beam (S1, S2, and S3) and one on the rebar on the other side (S4). The strain gages on the stirrups were mounted at one quarter of the leg distance from the bottom on side leg, and on the bottom leg. The strain gages on the stirrups were labeled as STL-1 and STL2 for SGs on vertical legs and STB-2 and STB-4 for SGs on bottom legs. Only two stirrups were instrumented with strain gages, and they were the first stirrups at the start of the splice zone from both sides. Lastly, five 60 mm (2.4 in.) SGs were placed on the upper part of the beams to measure the concrete compressive strains. These SGs were mounted at the locations corresponding to the rebar SG at the start of the splice zone (on one side) and its mid-length, on the top surface, 30 mm (1.18 in.), and 60 mm (2.36 in.) from the top of the beam, as shown in Fig. 5. All measuring devices were connected to an independent multiport data-acquisition system. Figs. 2, 3, and 4 give the reinforcement details for the beams with continuous bars and the beams with a splice length equal to 40 d_b_ [630 mm (25 in.)] with or without the presence of confining stirrups. A similar configuration was used for the beams with splice lengths of 60 and 80 d_b_. Fig. 5 and 6 show the details for the concrete SGs and potentiometer.

### Loading Protocol

The beams were tested under three cycles of four-point bending with a displacement control rate of 1.2 mm/min (0.047 in/min). The applied load increased monotonically until the first unloading at 1.15 times the computed cracking load (computed based on the notched cross section). The beam was reloaded again until it reached the calculated design load or until a rebar slipped 0.5 mm (0.02 in.), whichever came first. The beam was then unloaded and then loaded to failure. Loading and unloading were carried out to investigate slip accumulation during testing.

## Experimental Results and Discussion

Table 4 presents the results for all 16 specimens tested, including the values of moments obtained at failure (*M*_test_), the expected moment calculated based on material properties (*M*_th_) as per provision of ACI 440.11. The ratio of *M*_test_/*M*_th_ provides an indication of the capacity variation of specimens when changing splice lengths.

**Table 4:** Test results and failure mode

Beam ID	Splice length (*l*_d_) (mm)	Expected moment (Mth) (kN-m)	Test moment (Mtest) (kN-m)	Average test moment (kN-m)	Mid-span deflection (mm)	Mtest/Mth	Failure mode
G-C-00-1	Unspliced	153	149	149.5	115	0.97	Concrete crushing
G-C-00-2	Unspliced	153	150		112	0.98	Concrete crushing
G-40-00-1	630	163	59	66.0	50	0.36	Splitting
G-40-00-2	630	160	73		61	0.45	Splitting
G-60-00-1	950	163	77	84.0	57	0.47	Splitting
G-60-00-2	950	160	91		73	0.56	Splitting
G-80-00-1	1250	163	85	87.5	64	0.52	Splitting
G-80-00-2	1250	160	90		74	0.56	Splitting
G-40-100-1	630	165	95	98.5	81	0.58	**Slipping**
G-40-100-2	630	162	102		60	0.63	**Slipping**
G-40-200-1	630	165	81	79.0	69	0.49	Splitting
G-40-200-2	630	162	77		64	0.48	Splitting
G-60-100-1	950	165	132	132.0	117	0.8	**Slipping**
G-60-100-2	950	162	132		106	0.81	**Slipping**
G-60-200-1	950	164	104	102.0	86	0.63	Splitting
G-60-200-2	950	165	100		80	0.61	Splitting

### Flexural Behavior and Failure Modes

**Fig. 7:**
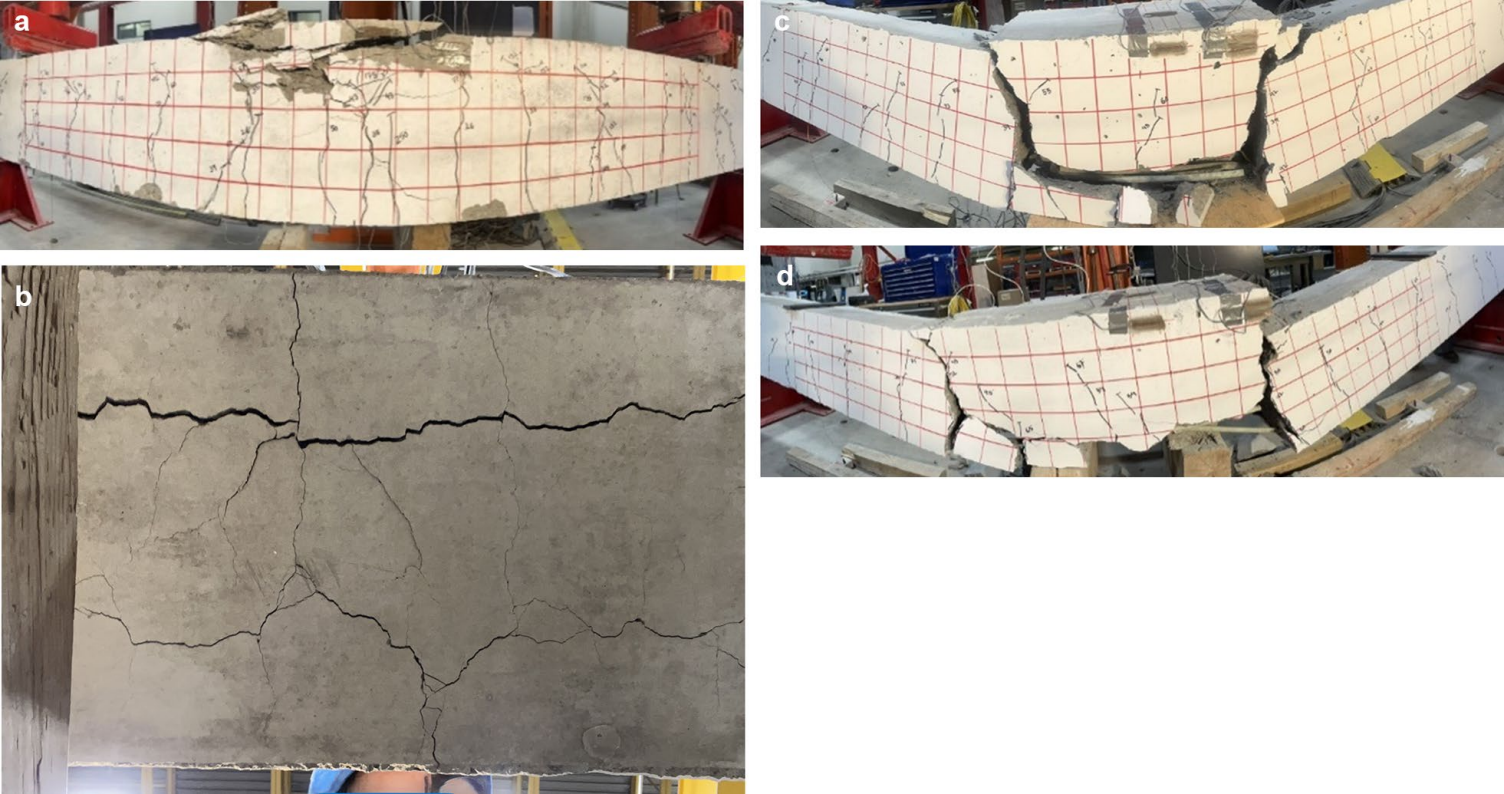
Modes of failure of the reference and spliced GFRP-RC beams without stirrups in the constant moment region

**Fig. 8:**
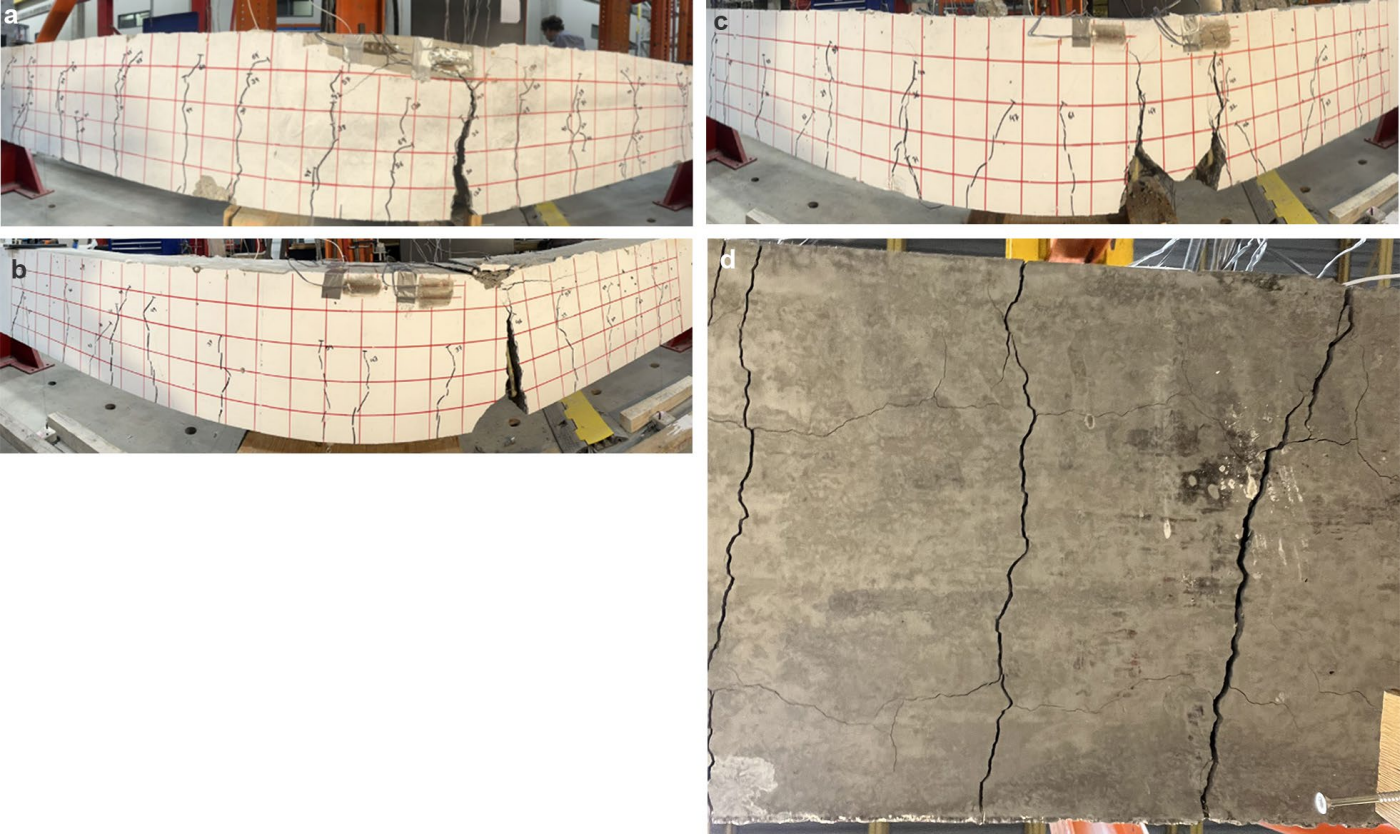
Modes of failure of the spliced GFRP-RC beams with stirrups in the constant moment region

Crack propagation was recorded during testing, as shown in Figs. 7 and 8. A 90 mm (3.5 in.) red grid in the constant moment zone serves as a reference in understanding crack propagation. Beams with continuous bars were notched at the same distance as beams with splice lengths equal to 40d_b_. Both beams in each configuration exhibited the same crack pattern, hence, only one is shown for reference.

#### Reference and Spliced GFRP-RC Beams Without Stirrups in the constant Moment Region

Fig. 7 presents the crack patterns in and failure modes of the reference and spliced beams without stirrups in the constant moment region. In the case of the reference beam (G-C-00), the first cracks developed simultaneously at the two notch locations at an average moment equal to 20 kN-m. The flexural cracks grew in number and width as the applied load increased. Longitudinal cracks also formed along the rebar on the bottom surface of the beam. When the moment reached 149 kN-m, many of the cracks reached near the top surface, causing the beams to fail by concrete crushing.

For G-40-00 and G-60-00, the cracks started at the location of notches and propagated vertically. Cracks also formed in-and-outside the splice zone; however, these cracks did not increase in their length and width significantly compared to cracks at notches. As the load increased, longitudinal cracks formed and started spreading along the rebar (Fig. 7b); the main cracks widened at the notches. The spreading of cracks on the bottom of the beam caused the entire concrete cover to split, as shown in Fig. 7c and d. Moreover, when the beams failed, they broke into parts without reaching the maximum expected capacities of either the concrete or GFRP. This indicates that the provided splice-length values were lower than needed for these materials to reach their ultimate capacities. The longitudinal cracks in G-80-00 were intersected by transverse cracks, forming a mesh of cracks on the bottom surface of the specimens. The beams failed when the cracks at the notch locations widened greatly and extended close to the beam’s top surface. A significant movement of rebar was evident upon postmortem observation.

#### Spliced Beams with Stirrups in the Constant Moment Region (G-40-100 to G-60-200)

Fig. 8a–d shows the mode of failure and crack propagation of the transversely confined spliced beams (G-40-100; G-40-200; G-60-100, and G-60-200). Unlike the beams without stirrups, the transversely confined beams exhibited higher capacity. Moreover, the presence of stirrups altered crack patterns and crack propagation. Similar to what occurred in the unconfined beams, the first cracks in these beams started at the notch locations, followed by other cracks inside and outside the lap-splice zone.

Fig. 8a shows that the crack at the notch in beam G-40–100 propagated vertically, reaching close to the top surface at failure. Longitudinal cracks also formed on the bottom surface of the beam in addition to transverse cracks. Unlike the case of the beams without stirrups, longitudinal cracks were intersected by stirrups. Therefore, the cracks formed mainly along the beam width and propagated vertically. Beam G-40-100 failed by bar slippage and a crack opening at the notch location. Rebar movement was observed after beam failure. The failure of beam G-40-200 with wider stirrup spacing (i.e., 200 mm) followed the same pattern, and failed by the concrete cover splitting between two stirrups at the start of the splice. The rebar moved significantly before the cover split. A comparison of beams G-40-00, G-40-100, and G-40-200 clearly shows that stirrup presence and spacing affected the cracking pattern and failure modes.

The cracks in G-60-100 initiated with the same pattern as the other specimens, that is, at the notch location. Longitudinal cracks were intercepted by stirrups. Each beam failed when the cracks at the notch propagated vertically, nearly reaching the top surface of the specimens. The potentiometers detached from the rebar due to its significant movement before failure. Concrete-cover detachment equal to the stirrup spacing was observed at the notch location, as shown in Fig. 8c. Wider and larger cracks were visible when the stirrup spacing was increased to 200 mm with the same splice length. Although longitudinal cracks were intercepted by stirrups, the stirrup spacing allowed the cracks to widen. This led to the detachment of the concrete between the stirrups. It should be noted that, after the first cracks at the notches, more cracks occurred outside the splice zone than within the splice length. This might be due to higher stiffness provided by a greater number of bars within the splice zone. Due to an abrupt change in stiffness just before the start of the spliced region and presence of the notch, cracks at this location were wider, and, in most specimens, failure occurred at this location. Fig. 8d shows that when stirrups were present cracks mostly propagated along width not length as otherwise shown in Fig. 7d.

### Moment–Deflection Curves

**Fig. 9:**
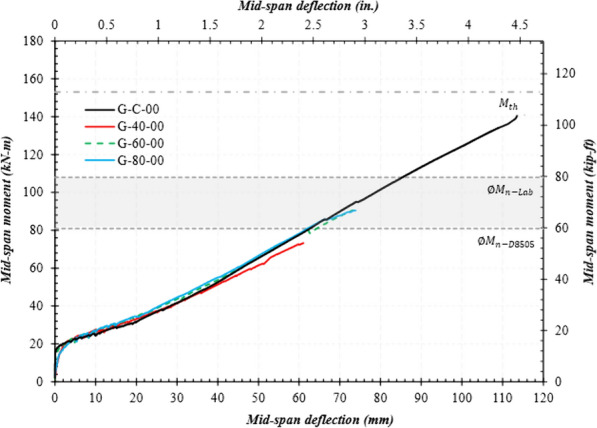
Mid-span moment vs. deflection of the reference and spliced beams without stirrups

**Fig. 10:**
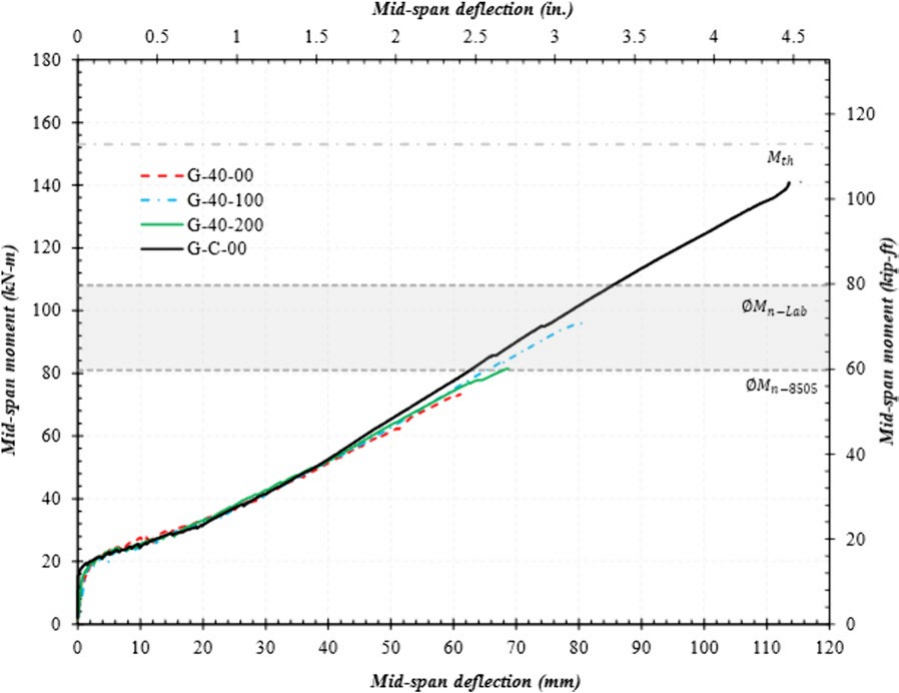
Mid-span moment vs. deflection of the specimens with a 60 d_b_ splice length

**Fig. 11:**
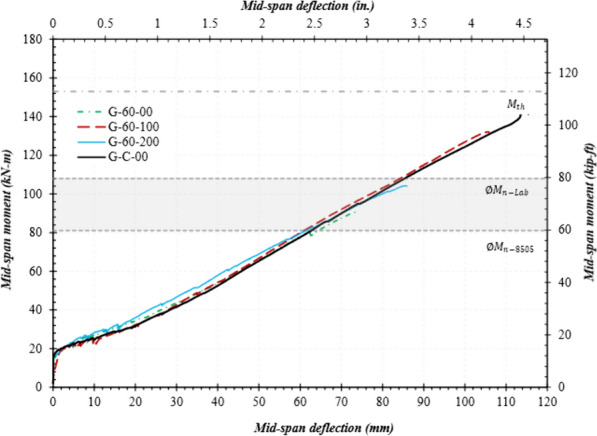
Mid-span moment vs. deflection of the specimens with a 60 d_b_ splice length

Figs. 9, 10, and 11 present the mid-span moment vs. deflection of reference and spliced GFRP-RC beams. Also shown are the lines for the expected ultimate moment and design moment calculated using actual material properties. *ΦM*_n_-lab represents the moment capacity using actual material properties, whereas *ΦM*_n_-D8505 represents that calculated as per ASTM D8505. In the graphs, only the average of each beam configuration is presented, and the unloading stages of the loading pattern were also removed. All moment values discussed in this section are the average of two specimens with the same splice length as provided in column 5 of Table 4.

#### Reference and Spliced GFRP-RC beams Without Stirrups

Fig. 9 presents the mid-span moment vs. deflection of the reference and spliced (i.e., 40, 60, and 80 d_b_) GFRP-RC beams without stirrups in the constant moment region. At low magnitudes of moment (i.e., 20 kN-m), the beams remain uncracked, as is evident from the high stiffness values. When concrete cracking occurred, it led to small drops in load and a significant reduction in stiffness. All the beams had bilinear behavior up to failure. The failure moment for the reference beams was equal to 149 kN-m at a deflection equal to 113 mm, which is slightly less than the expected ultimate moment (i.e., 153 kN-m). Finally, the reference specimens failed by concrete crushing, as shown in Fig. 7.

The failure moment for a specimen with a splice length equal to 40 d_b_ (G-40-00) was 66 kN-m, which is 56% lower than the reference beam (i.e., 149 kN-m). The specimen failed without reaching either the expected or design moment values. The 60d_b_ specimen (G-60-00) showed slightly higher resistance to the applied loads and failed after reaching a moment value 27% higher than G-40-00 but still its capacity was 44% lower than the reference beam (i.e., 84 kN-m vs. 149 kN-m).

Interestingly, the beams with lap-splice lengths equal to 80d_b_ did not show, on average, much difference in capacity than the specimens with the 60 d_b_ splice length (88 kN-m vs. 84 kN-m, respectively). This indicates that the capacity of the spliced beams did not increase in proportion to the length of the lap splice. This observation is consistent with those made by Mosely et al. (2008).

#### Spliced GFRP-RC Beams with Stirrups in the Constant Moment Region

Fig. 10 shows the beams with a lap-splice length equal to 40d_b_ and stirrups placed on 100 mm (4 in.) or 200 mm (8 in.) centers. As evident in the figure, the stirrups had a substantial effect on specimen capacity. For example, G-40-100 reached a moment at failure equal to 99 kN-m, which is 50% higher than that without stirrups (G-40-00). Similar behavior was observed for the G-40-200 specimens. As expected, however, beam capacity decreased as the stirrup spacing increased. For example, G-40-200 reached a moment equal to 79 kN-m, which is 21% lower than G-40-100. This implies that both stirrup presence and their spacing significantly affected beam capacity. Similar observations were reported in the literature when increasing the stirrup spacing reduced the capacity of the GFRP-RC beams (Wu et al., 2022).

Fig. 11 presents the values of the mid-span moment vs. deflection for the beams with a 60 d_b_ lap-splice length. Beam G-60-100 achieved a capacity that was only 11% lower than that of the reference specimen (G-C-00) and 57% higher than the one without stirrups in the spliced region (G-60-00). It should be noted that the provided lap-splice length (i.e., 60 d_b_) is much less than that required in ACI 440.11 (i.e., 102 d_b_). This implies that providing a 102 d_b_ splice length as required in ACI 440.11 would have greatly penalized the design when stirrups were used. The beams with splice lengths equal to 60 d_b_, with stirrups at 200 mm (G-60-200) only reached 62% of the expected capacity, but it was 21% higher than the one without stirrups. For example, G-60-200 achieved a moment capacity equal to 102 kN-m, as compared to 84 kN-m for G-60-00.

The above discussion clearly shows that stirrups substantially affected beam capacity. The splitting cracks that generated along the rebar were intercepted by stirrups. As Figs. 7 and 8 show, the presence of stirrups changed the failure mode from splitting to slippage. The confinement provided by the stirrups and concrete cover resisted the splitting forces generated after a loss of chemical bond. When the spacing was increased, the cracks that formed between the stirrup legs had sufficient space to widen and cause the cover to split. The manufacturing process causes the inner bend of stirrups to be smooth and slippery, offering less slip resistance than conventional steel stirrups. Therefore, when the splitting component of the bond force was exceeded by stirrup confinement, its tangential component produced slippage of the GFRP rebars, causing the specimens to fail. This mechanism delayed the failure and increased the overall capacity of the specimens.

### Mid-span Moment vs. Slip

**Fig. 12:**
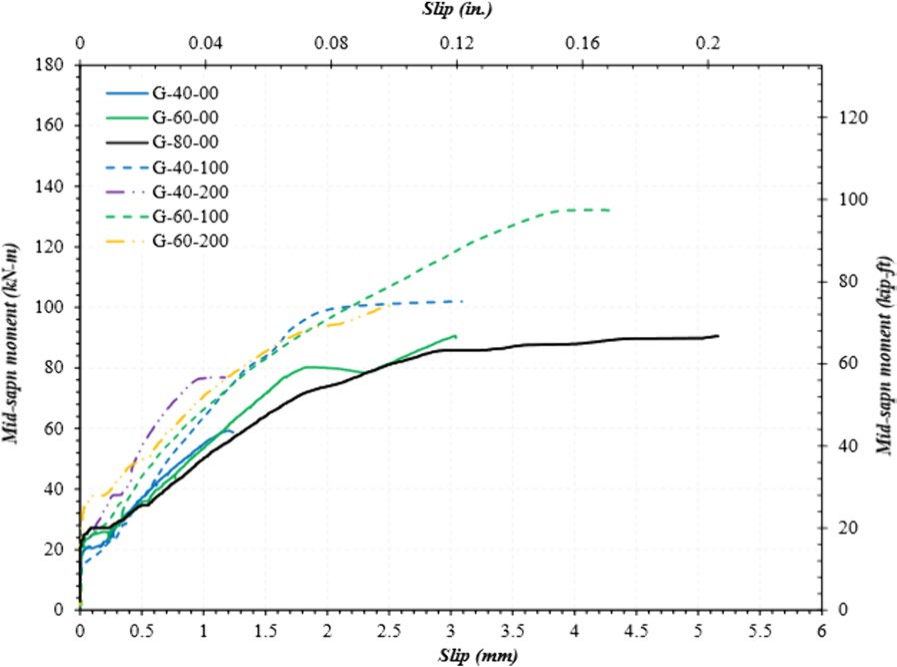
Mid-span moment vs. slip

As evident in Fig. 12, the rebars in all the specimens started slipping upon initial cracking. After the initial cracking, the rebar kept moving but at a significantly lower rate. This indicates that the rebars locked again and resisted slippage. Consequently, it could be concluded that the rebars lost their mechanical interlock with the concrete at the cracking moment. A bond remained between the two materials, however, due to friction. Since friction depends on rebar surface area, the specimens with longer lap-spliced lengths exhibited higher slip resistance and ultimately achieved higher capacity before failure.

The rebars in the specimens with stirrups showed more slip resistance than those without stirrups. A rebar in a G-60-00 specimen slipped 1.18 mm (0.046 in.) at a moment of 60 kN-m. In contrast, a rebar in a G-60-100 specimen experienced a slip of only 0.87 mm (0.034 in.) at the same moment, which equates to a 27% reduction in slip. The lower slip value impacted the specimen’s overall capacity (84 kN-m vs. 132 kN-m). Therefore, it can be concluded that stirrups increased the confinement and resistance to the splitting of the concrete cover and to rebar slippage. As a result, the beams with stirrups exhibited higher capacity than those without stirrups. Note: some potentiometers failed during casting or testing. Consequently, Fig. 12 shows only those potentiometers in agreement with the experimental moments.

### Mid-span Moment vs. Rebar Strain

**Fig. 13:**
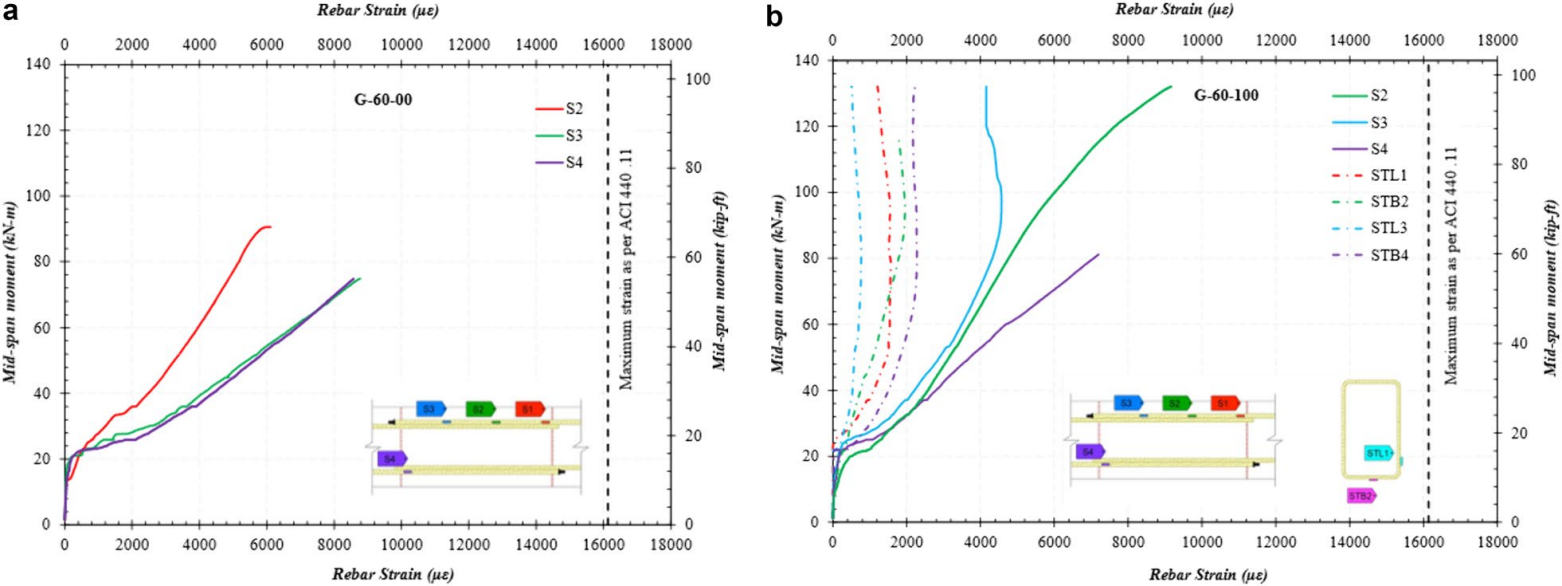
Strain variation in GFRP rebars along the splice length and in stirrups

Fig. 13 presents a typical mid-span moment vs. rebar strain in the specimens G-60-00 and G-60-100. As evident in Fig. 13, SGs did not record strain values up to cracking. Thereafter, an abrupt increment in strain can be noticed. This shows that in an uncracked specimen, all the loads are taken by concrete cross section. At the onset of cracking, stresses are completely transferred to the GFRP rebar at the location of cracks (as evident by a jump in strain values; see Fig. 13). However, between cracked locations, these stresses are transferred back to the concrete. Hence, an in-and-out bond stress situation exists between cracked and uncracked regions. This is evident by different strain values in SGs along the splice length (*S2, S3, and S4; Note: S1 failed during casting in mentioned specimens*). As cracks grew in number and length along the splice, it can be expected that the stresses between the cracks become uniform since there is not sufficient length to transfer them back to the concrete. This observation was made by closely monitoring the linearity of strain curves after certain values of loads.

Strain gages were installed on the rebars to monitor strain variation throughout the test to analyze stress transfer along the lap-splice length and to measure the maximum tensile stress before failure. Many of the strain gages stopped working or failed, however, during specimen testing. Consequently, rebar strain was also calculated using the maximum moment to verify the strain recorded by the SGs. This method has been used and verified by other researchers (Aly et al., 2006; Esfahani et al., 2013; Harajli & Abouniaj, 2010; Mosely et al., 2008; Tighiouart & Benmokrane, 1999). Table 5 gives both the calculated strain using moments and recorded with strain gages.

**Table 5:** Bond strengths of the GFRP-RC specimens

Beam ID	Splice length (*l*_*d*_) (mm)	Mtest (kN-m)	Recorded strain (*ε*_*r*_)	*M*_test_ strain (*ε*_*M*-test_)	Stress (MPa)	Average bond strength (*μ*_avg-test_) (MPa)
G-C-00-1	Un-spliced	149	0.0084	0.0151	980	–
G-C-00-2	Un-spliced	150	0.0092	0.0151	980	–
G-40-00-1	630	59	0.0064	0.0060	383	2.41
G-40-00-2	630	73	0.0089	0.0074	480	3.02
G-60-00-1	950	77	0.0080	0.0078	506	2.11
G-60-00-2	950	91	0.0091	0.0090	590	2.46
G-80-00-1	1250	85	0.0092	0.0085	551	1.75
G-80-00-2	1250	90	0.0099	0.0091	591	1.88
G-40-100-1	630	95	0.0083	0.0099	643	4.05
G-40-100-2	630	102	–	0.0106	688	4.33
G-40-200-1	630	81	0.0075	0.0085	552	3.48
G-40-200-2	630	77	0.0082	0.0080	520	3.28
G-60-100-1	950	132	0.0107	0.0138	896	3.74
G-60-100-2	950	132	0.0091	0.0138	897	3.75
G-60-200-1	950	104	0.0077	0.0109	707	2.92
G-60-200-2	950	100	0.0105	0.0105	682	2.85

Recorded strain values are those measured with SG’s and *M*_test_ strain are those measured using experimental moment

The strain in the rebars in the reference beams at failure was equal to 0.0151 which is less than the ultimate design strain of GFRP rebars (i.e., *C*_*E*_* x ε*_fu_ = 0.0189), as the specimen failed by concrete crushing. In the case of specimens with 40 d_b_, 60 d_b_, and 80 d_b_ splice lengths without stirrups in the constant moment region, the strain values were much lower than expected at failure both in the concrete and GFRP. The strain values calculated using maximum moments for these specimens at failure were equal to 0.0067, 0.0084, and 0.0095 vs. 0.0189 (i.e., average values). The strain in the concrete was also lower than 0.003, signifying bond failure.

The strain values were higher for the GFRP rebars confined with stirrups in the constant moment region. The strain calculated in G-40-100 was equal to 0.0106 compared to 0.0067 for G-40-00. This holds true as well for beams with the 60d_b_ splice length with stirrups every 100 mm (8 in.), where calculated strain was equal to 0.0138 compared to 0.0084 for beams without stirrups. Hence, the presence of stirrups helped GFRP bars provide more resistance to applied stresses. The strain values were lower when the stirrup spacing was increased to 200 mm (8 in.). For example, the strain values for G-60-100 and G-60-200 were equal to 0.0138 and 0.0107, respectively. The strain values discussed above are an average of two specimens.

Fig. 13b shows that, up to the cracking of the beam, the strain in the stirrups remained negligible. Nonetheless, as the load increased, the strain in the stirrups trended upward, signifying their involvement in resisting splitting forces. The maximum strain values in the stirrups were observed at close to the peak load with the bottom leg showing more strain than the sides. After a certain value, the strain in GFRP stirrups stabilized. This might indicate that the splitting stresses were exceeded by the resistance provided by stirrups, and the bars started slipping at a higher rate under the tangential component of force. This observation might be validated by comparing curves at a moment of 80 kN-m in Figs. 12 and 13. The magnitude of strain in the stirrups indicates that the confining stirrups resisted the expansion of the concrete in the lap-spliced area, thereby improving the bond.

### Average Bond Strength of Specimens

The average bond strength (*μ*_test_) was determined by calculating the stress in the reinforcement at failure as provided in Table 5. The bar stress for all specimens was calculated from the recorded maximum moment as used and verified by other researchers (Esfahani et al., 2013). This method for calculating the bar stress was adopted to rectify any errors in data collection using SGs. The tensile bar stress was calculated using Eq. 2.

2 $${f}_{s}=\frac{{M}_{\text{test}}}{{A}_{f}jd}$$

where *M*_test_ is the maximum moment at failure of the specimen (kN-m), *A*_*f*_ is the cross-sectional area of all spliced tensile reinforcing bars (m^2^), and *jd* is the resistant moment arm (mm). The value of *jd* was calculated based on the Todeschini stress–strain model.

The average bond stress was calculated as:

3 $${u}_{\text{test}}=\frac{{d}_{b}{f}_{s}}{4{l}_{e}}$$

where *f*_*s*_ is the stress developed in the reinforcement (MPa), *d*_*b*_ is the diameter of the longitudinal reinforcement (mm), and *l*_*e*_ is the embedment length (mm).

**Fig. 14:**
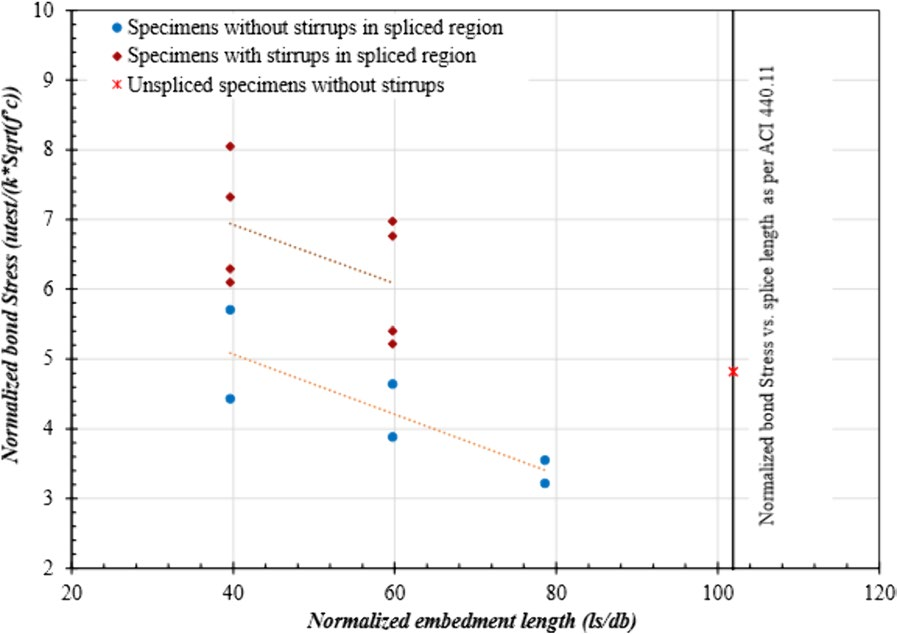
Normalized bond stress vs. lap-splice length

Table 5 shows that the average bond stress calculated from the maximum moments at failure did not follow a linear pattern. This might be due to the non-uniform distribution of bond stresses as the formation of cracks affects in-and-out bond stresses. The formation of cracks at varying locations along the rebars with different spliced lengths resulted in different bonded lengths transferring stresses back to the concrete. This affected the variation in stresses along the rebar. Fig. 14 presents the curve of normalized bond stress versus normalized lap-splice length. To account for the variability in concrete compressive strength for different specimens, as given in Table 2, the bond-strength values were divided by the square root of the concrete compressive strength. The “*ĸ*” factor adjusts for the unit system used, with a value of 1 for the inch–pound system and 0.083 for SI units. Fig. 14 shows that the bond strength decreased as the normalized spliced length increased. Moreover, the effect of presence of stirrups on the bond strength is clearly visible in Fig. 14.

### Comparisons of Bond Strength with Different Approaches

#### ACI 440.11 Bond-Strength Equation (ACI 2022)

Wambeke and Shield (2006) used an approach similar to that of Orangun et al. (1977) and proposed an equation for calculating the bond strength of GFRP bars for the splitting mode of bond failure as given below:

4 $$\frac{u}{{0.083\sqrt {f^{\prime}_{c} } }} = \frac{1}{\alpha }\left( {4.0 + 0.3\frac{C}{{d_{b} }} + 100\frac{{d_{b} }}{{l_{d} }}} \right)$$

where *u* is the bond strength in MPa; *f’*_*c*_ is the concrete compressive strength in MPa; *C* is the lesser of the cover to the center of the bar or one-half of the center-to-center spacing of the bars being developed in mm; *d*_*b*_ is the bar diameter in mm; *l*_*d*_ is the embedment length of the reinforcement inside the concrete in mm; and *α* is the bar location modification factor, taken as equal to 1.3 if more than 300 mm (12 in.) of concrete is cast below otherwise equal to 1.0.

The equation was endorsed by ACI Committee 440 and became part of ACI 440.1R-15. The equation does not, however, include the effect of stirrups on the bond strength.

### CSA S806:12 Bond-Strength Equation (CSA (Canadian Standard Association), 2012)

Similar to ACI 440.11, CSA S806:12 disregards the effect of confinement provided by stirrups on the bond strength. It does take into consideration the different reinforcement surface treatments. The bond-strength equation as provided in chapter 9 of CSA-S806:12 is given below as Eq. 5.

5 $$\mu_{S806} = \frac{{d_{cs} \sqrt {f^{\prime}_{c} } }}{{1.15\left( {k_{1} k_{2} k_{3} k_{4} k_{5} } \right)\pi d_{b} }}{ }$$

where *d*_*cs*_ is the smaller of the distance from the center of the bar being developed to the closest concrete surface or two-third of the center-to-center spacing of the bars being developed and shall not exceed 2.5 d_b_; *k*_*1*_ is the bar location factor (1.3 when more than 300 mm (12 in.) of fresh concrete is placed below horizontal reinforcement, 1.0 for all other cases); *k*_*2*_ is the concrete density factor (1.3 for structural low-density concrete, 1.2 for structural semi-low-density concrete, 1.0 for normal-density concrete); *k*_*3*_ is the bar-size factor (0.8 for *A*_*b*_ ≤ 300 mm^2^, 1.0 for *A*_*b*_ > 300 mm^2^);* A*_*b*_ is the area of an individual bar; *k*_*4*_ is the bar fiber factor (1.0 for CFRP and GFRP, 1.25 for AFRP); and *k*_*5*_ is a factor to consider bar surface (1.0 for surface roughened, sand-coated, or braided surfaces, 1.05 for spiral pattern surfaces or ribbed surfaces, and 1.8 for indented surfaces).

### CSA S6:19 bond-strength equation (CSA S6:19, 2019)

Unlike ACI 440.11 and CSA S806:12, the *Canadian Highway Bridge Design Code* (CSA S6:19) provides guidelines to include the effect of stirrups on the bond strength. The bond-strength equation in CSA S6:19 is provided below as Eq. 6.

6 $${\mu }_{S6-19}=\frac{{f}_{cr}\left({d}_{cs}+{K}_{\text{tr}}{E}_{frp}/{E}_{s}\right)}{0.45\pi {d}_{b}{k}_{1}{k}_{4}}\text{ where}\quad { K}_{\text{tr}}=\frac{{A}_{\text{tr}}{f}_{y}}{10.5sn}$$

where *f*_*cr*_ is the concrete cracking strength ($0.45\sqrt{{f{\prime}}_{c}}$) in MPa; *K*_tr_ is the transverse reinforcement index, representing the contribution of confining reinforcement; *E*_*frp*_ and *E*_*s*_ denote the elastic modulus of FRP and steel bars in GPa, respectively; *f*_*y*_ is the yield strength of transverse reinforcement in MPa; *s* is the maximum center-to-center spacing of the transverse reinforcement within the embedment length; *n* is the number of bars being developed along the potential plane of splitting; *k*_*1*_ is the bar location factor (1.3 when more than 300 mm (12 in.) of fresh concrete is placed below horizontal reinforcement, 1.0 for all other cases); and *k*_*4*_ is the bar surface factor, defined as the ratio of the bond strength of the FRP bar to that of a deformed steel bar with the same cross-sectional area as the FRP bar but should not exceed 1.0. In the absence of experimental data, *k*_*4*_ shall be taken as 0.8. The term in brackets in numerator (i.e., *d*_cs_ + *K*_*tr*_* x E*_frp_*/E*_*s*_) should not be taken greater than 2.5 d_b_.

### CSA S6:25 bond-strength equation (CSA S6:25, 2025)

The upcoming edition of CSA S6:25 (CSA S6:25, 2025) proposes a modified equation for predicting the bond strength of GFRP-RC members. In this equation, *k*_*4*_ (i.e., bar surface factor) has been moved to the numerator in the bond-strength equation, and *K*_tr_ has been updated by incorporating a value for a *f*_*y*_ of 400 MPa. The limitations on terms within the brackets in the numerator (i.e., *d*_cs_ + *K*_tr_* x E*_frp_*/E*_s_) remain the same as in CSA S6:19. The updated equation is provided below as Eq. 7.

7 $${\mu }_{S6-25}=\frac{{k}_{4}{f}_{cr}\left({d}_{cs}+{K}_{tr}{E}_{frp}/{E}_{s}\right)}{0.45{k}_{1}\pi {d}_{b}}\text{ where}\quad {K}_{\text{tr}}=\frac{38{A}_{tr}}{sn}$$

### 5Bond-strength equation as proposed in the literature (Esfahani et al., 2013)

There are many equations in the literature for calculating the bond strength of FRP bars. In this study, an equation proposed by Esfahani et al. (2013) was opted as it closely aligns with the aims of this study. Esfahani et al. (2013) proposed updates on the bond-strength equation developed by Wambeke and Shield (2006) and proposed an additional term to include the effect of transverse reinforcement. The additional term is similar to that proposed by Orangun et al. (1977). Since GFRP reinforcement has different surface treatments, a factor (*f*_*R*_) depending on the reinforcement surface was also introduced to the additional term, signifying the different surfaces of GFRP bars. Table 6 provides numerical values of *f*_*R*_ for different surface treatments as proposed by Esfahani et al. (2013). The equations proposed by Esfahani for the bond strength of FRP bars without and with the presence of stirrups are provided below as Eqs. 8a, and 8b.

8a $$\frac{u}{{0.083\sqrt {f^{\prime}_{c} } }} = \frac{1}{\alpha }\left( {2.36 + 0.177\frac{C}{{d_{b} }} + 59\frac{{d_{b} }}{{l_{d} }}} \right){ }$$

8b $$\frac{u}{{0.083\sqrt {f^{\prime}} }} = \frac{1}{\alpha }\left( {2.36 + 0.177\frac{C}{{d_{b} }} + 59\frac{{d_{b} }}{{L_{d} }} + f_{R} \frac{{A_{tr} f_{yt} }}{{sd_{b} }}} \right){ }$$

where *A*_tr_ is the area of the transverse reinforcement in mm^2^; *s* is the spacing between stirrups in mm*, f*_*y*t_ is the yield strength of the transverse reinforcement in MPa; and *f*_*R*_ is the factor signifying the reinforcement type.

**Table 6:** Numerical values of the *f*_*R*_ factor (Esfahani et al., 2013)

Surface properties	*f*_*R*_
Helically wrapped	0.03
Indented	0.08
Sand coated	0.17
Ribbed	0.21

**Fig. 15:**
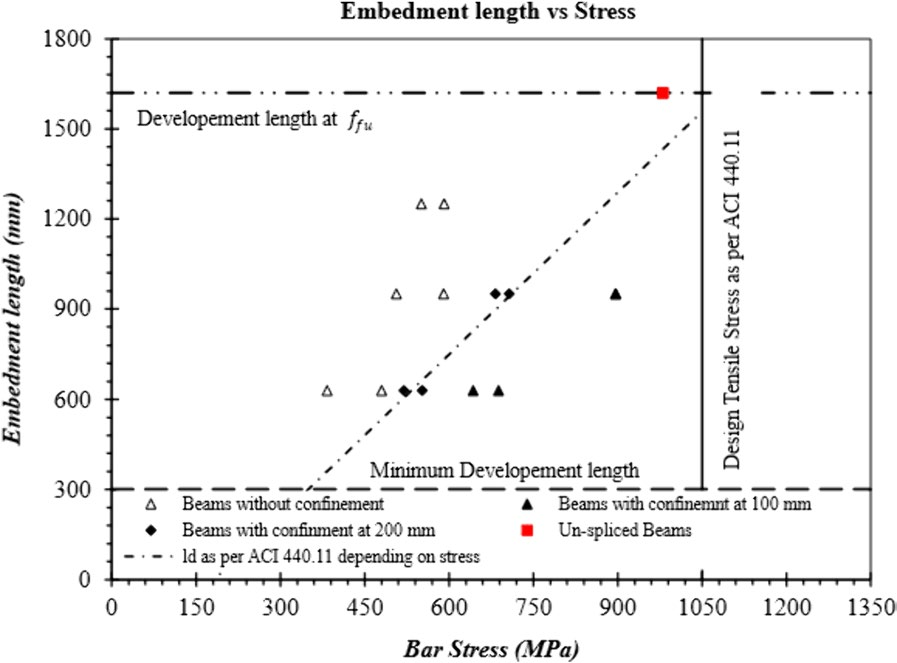
Bond strength of the GFRP-RC beams with and without confining stirrups

A graph of Embedment length vs. longitudinal stress in the bar is shown in Fig. 15. The dotted inclined line shows minimum development length required as per ACI 440.11 at corresponding stress. It further shows that at these values of embedment lengths, the specimen should have reached the bar stress shown in abscissa of the graph. It can be observed in Fig. 15, that calculated bond stresses for most beams without stirrups or stirrups at 200 mm are lower than expected at provided embedment length. However, in the presence of stirrups at 100 mm on centers, the specimens reached the corresponding required stress before failure. Red color highlights reference beam failed by concrete crushing. Comparing specimens with and without stirrups at the same spliced lengths highlights the contribution of stirrups toward strength. Fig. 15 further highlights that, a lower factor than the currently specified 0.85 (environment reduction factor; *C*_*E*_) could be employed on *f*_fr_ in current development length equation to avoid longer development length, when only a particular stress is desired, and stirrups are present. For example, if *C*_*E*_ was equal to 0.70 instead of 0.85, corresponding design stress would be 864 MPa, whereas G-60-100 reached a stress value equal to 897 MPa before failure. It should be remembered that embedment length for G-60-100 in the current study is 41% lower than required as per ACI 440.11.

As can be observed in Table 7, predicted values calculated using Eq. 4 are generally larger than experimental data except for specimens with closely spaced stirrups. The average test-to-predicted ratio when using Eq. 4 is equal to 0.80, and the standard deviation is 0.17. Therefore, it can be concluded that the ACI 440.11 equation overestimates the bond strength for specimens without stirrups. However, it yielded conservative results for specimens with closely spaced stirrups. This observation is in line with the findings of Esfahani et al (2013).

**Table 7:** Experimental and predicted bond-strength values

ID	*u*_test_ (MPa)	*u*_ACI_ (MPa)	*u*_CSA-S806_ (MPa)	*u*_CSA-S6:19_ (MPa)	*u*_CSA-S6:25_ (MPa)	Esfahani et al. (2013)	*u*_test_*/u*_prd_ ACI 440.11 (Eq. 4)	*u*_test_*/u*_prd_ *u*_CSA-S806_	*u*_test_*/u*_prd_ *u*_CSA-S6:19_	*u*_test_*/u*_prd_ *u*_CSA-S6:25_	*u*_*test*_*/u*_*prd*_ Esfahani (2013)	*u*_test_*/u*_prd_ (Eqs. 8a and 8b) with *f*_*R*_ = *0.11*
C-00-1	2.4	2.91	4.3	5.97	3.82	1.66	0.82	0.56	0.4	0.63	1.27	1.27
C-00-2	2.4	2.91	4.3	5.97	3.82	1.66	0.82	0.56	0.4	0.63	1.28	1.28
40-00-1	2.41	4.01	4.3	6.51	4.17	2.37	0.6	0.56	0.37	0.58	1.12	1.12
40-00-2	3.02	3.91	4.3	6.34	4.06	2.3	0.77	0.7	0.48	0.74	1.42	1.42
60-00-1	2.11	3.6	4.3	6.51	4.17	2.09	0.59	0.49	0.32	0.51	1.08	1.08
60-00-2	2.46	3.46	4.3	6.34	4.06	2.04	0.71	0.57	0.39	0.61	1.30	1.30
80-00-1	1.75	3.3	4.3	6.51	4.17	1.96	0.53	0.41	0.27	0.42	0.97	0.97
80-00-2	1.88	3.25	4.3	6.34	4.06	1.92	0.58	0.44	0.3	0.46	1.06	1.06
40-100-1	4.05	4.08	4.3	6.63	4.24	6.06	0.99	0.94	0.61	0.95	0.71	1.09
40-100-2	4.33	3.97	4.3	6.44	4.12	5.9	1.09	1.01	0.67	1.05	0.78	1.1
40-200-1	3.48	4.08	4.3	6.63	4.24	4.23	0.85	0.81	0.52	0.82	0.79	1.0
40-200-2	3.28	3.97	4.3	6.44	4.12	4.12	0.83	0.76	0.51	0.79	0.83	1.05
60-100-1	3.74	3.61	4.3	6.63	4.24	5.79	1.04	0.87	0.56	0.88	0.68	0.97
60-100-2	3.75	3.51	4.3	6.44	4.12	5.63	1.07	0.87	0.58	0.91	0.70	1.01
60-200-1	2.92	3.57	4.3	6.54	4.19	3.91	0.82	0.68	0.45	0.70	0.79	1.02
60-200-2	2.85	3.57	4.3	6.54	4.19	3.91	0.8	0.66	0.44	0.68	0.76	0.98
Mean							0.80	0.68	0.45	0.71	0.97	1.10
STD							0.17	0.18	0.11	0.18	0.25	0.14

CSA S806:12 also overestimated the bond strength with a test-to-predicted ratio equal to 0.68 and a standard deviation of 0.18. It is worth noting that CSA S806:12 yielded constant bond-strength values for all the specimens. This is due to the maximum limit on the value of the square root of concrete strength as it cannot be taken greater than 5 MPa. In this study, all the values of the square root of the concrete strength were greater than 5. Since its value cannot be greater than the stated limit in the code, √*f’*_*c*_ was taken as equal to 5 to calculate bond strengths for all specimens. In addition, the value of *d*_*cs*_ should not be greater than 2.5d_b_. With constant concrete cover [i.e., 38 mm (1.5 in.)] and only one bar diameter [i.e., 15.9 mm (No. 5)], *d*_cs_ was calculated equal to 45.95 mm (1.8 in.), whereas 2.5d_b_ was equal to 39.75 (1.56 in.). Hence, a constant value equal to 39.75 mm (1.56 in.) was adopted. The remaining constant parameters of *k*_*1*_ to *k*_*5*_ were equal to 1.0 for this study. Hence, the resulting values of bond strength were constant in all the specimens, as shown in Table 7. The test-to-predicted ratio of bond strengths for all the specimens was below unity except for those with stirrups on 100 mm (4 in.) centers. This was even though CSA S806:12 disregards the contribution of stirrups to the bond strength. The contribution of stirrups is evident, however, when comparing test-to-predicted ratios for CSA S806:12, implying that the code conservatively ignored the effect of stirrup confinement on bond strength.

CSA S6:19 provides guidelines to include the effect of confining stirrups on the bond strength. The expression for bond strength, however, significantly overestimates the bond strength of GFRP bars. Therefore, even in the presence of stirrups, the bond strength of the specimens was significantly lower than that predicted with Eq. 6. CSA S6:19 limits the maximum combined contribution of *d*_cs_ + *K*_tr_* E*_*f*_*/E*_*s*_ to not greater than 2.5 d_b_ (i.e., 39.75 mm (1.56 in.). Most codes, however, require that beams be designed with a minimum cover equal to 38 mm (1.5 in). In the presence of M16 (No. 5) bars (as in this study), the contribution of stirrups will be overshadowed by this limit. The test-to-predicted ratio for beam specimens according to CSA S6:19 is equal to 0.45, and the standard deviation is 0.11, as provided in Table 7.

Table 7 provides the bond-strength values calculated with the updated equation in CSA S6:25 (CSA S6:25, 2025). The figure shows that Eq. 7 predicted bond-strength values closer to experimental results than CSA S6:19, and CSA S806:12. The average test-to-predicted ratio was equal to 0.7 and a standard deviation 0.18. The *k*_*1*_ (i.e., bar location factor) was taken equal to 1.0 for this study, and *k*_*4*_ (i.e., bar surface factor) for sand-coated bars was equal to 0.8. The maximum limit equal to 2.5d_b_ on the term in the numerator (i.e., *d*_cs_ + *K*_tr_* x E*_frp_*/E*_*s*_) controlled for all specimens with or without stirrups. Hence, the only variable that affected bond strength was concrete strength. Similar to CSA S806:12 and CSA S6:19, the new proposed equation for bond strength in CSA S6:25 does not depend on embedment length; the calculated bond strength only slightly varied for all the specimens depending on concrete strength. Note that the test-to-predicted ratio for the specimens with closely spaced stirrups (i.e., 100 mm (4 in.) c/c) is equal to 0.95 with a standard deviation of 0.074. Hence, the newly adopted equation in CSA S6:25 better predicted the bond strength for the specimens with closely spaced stirrups, even though upper limit on confinement contribution still hold.

**Fig. 16:**
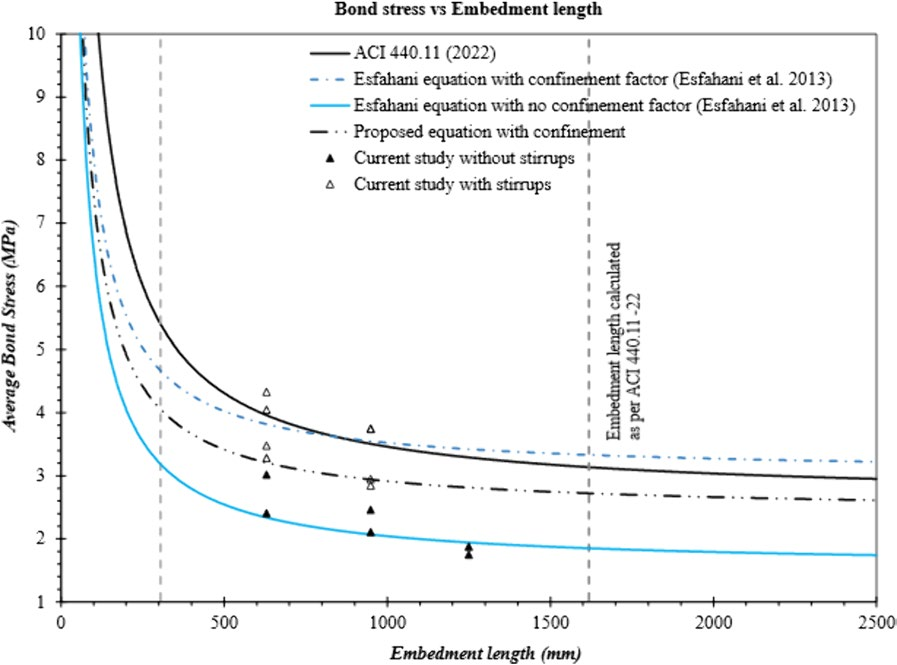
Bond strength of the GFRP-RC beams with and without confining stirrups

Similarly, bond strengths were predicted with the equations proposed by Esfahani et al. (2013). Table 7 shows that, by examining the test-to-predicted ratio of specimens without stirrups, Eq. 8a accurately predicted the bond strength with a test-to-predicted ratio equal to 1.18 and a standard deviation of 0.15 (specimens without stirrups). In addition, most of the test data falls above the line of Eq. 8a, as shown in Fig. 16. Nevertheless, Eq. 8b overestimated the bond strength with an average test-to-predicted ratio equal to 0.75 and a standard deviation of 0.05 (only for the specimens with stirrups). Only three beams with closely spaced stirrups had a bond strength higher than the value predicted with Eq. 8b. This might be due to the fact that the findings of Esfahani et al. (2013) were based on steel stirrups, whereas this study used GFRP stirrups. The difference in stiffness and the manufacturing process of the GFRP stirrups might have influenced the results.

The factor *f*_*R*_ proposed for sand-coated bars was based on the test data of the authors own experiments, which resulted in pullout failures due to very short embedment lengths, and the experimental results reported by Aly et al. (2006), in which steel stirrups were used. Hence, the bond-strength equation proposed by Esfahani et al. (2013) for sand-coated bars in the presence of stirrups mainly depends on the findings of Aly et al. (2006). In order to evaluate the findings in this study, an *f*_*R*_ for sand-coated bars equal to 0.11 were used in Eq. 8b, and the equation accurately predicted the bond strength. Table 7 shows that the test-to-predicted ratio when using the updated coefficient *f*_*R*_ in Eq. 8b was equal to 1.1 and had a standard deviation equal to 0.14 (*f*_*R*_ = 0.11). Fig. 16 provides a comparison of the bond stresses determined with different bond-strength equations.

As stated in Sect. 3.3, the amount of slip decreased with the use of stirrups, so steel stirrups with a high relative rib area might provide more resistance to slip, thereby increasing the bond strength. For practical purposes, however, steel stirrups are unlikely to be used with longitudinal GFRP reinforcement. Given that, an update to surface modification factor *f*_*R*_ is needed. Due to the limited experimental data available on transversely confined sand-coated GFRP reinforcement, a modification to *f*_*R*_ equal to 0.11 is proposed based on the reported study.

**Fig. 17:**
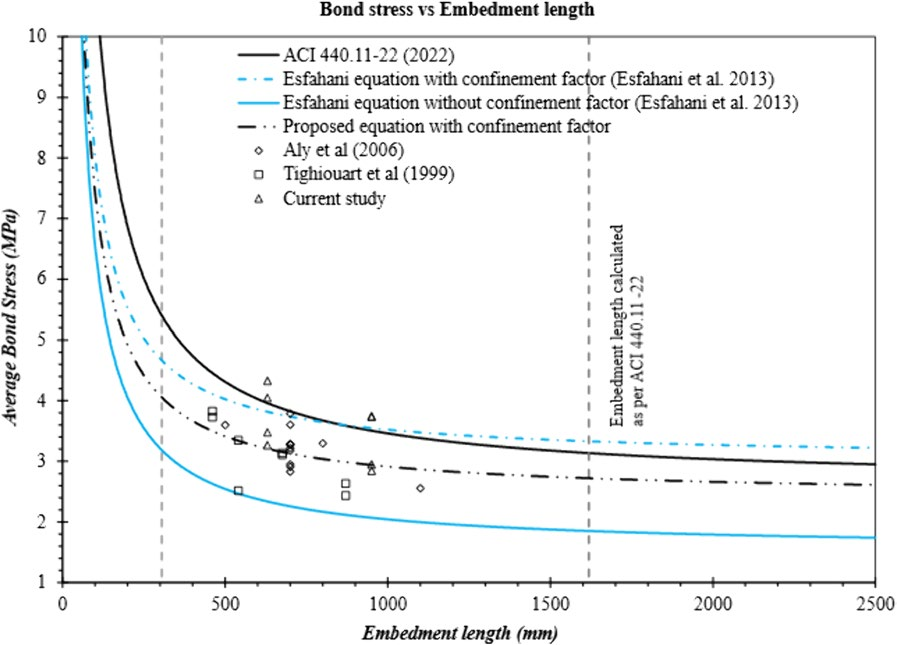
Bond strength of the GFRP-RC beams with confining stirrups

Fig. 17 provides the test results used by Esfahani et al. (2013) to propose a bond-strength equation and the experimental results from current study. As shown, most of the bond-strength values fall close to the line for the updated coefficient (*f*_*R*_) for sand-coated bars in the equation proposed by Esfahani et al. (2013). Hence, the bond-strength equation proposed by Esfahani et al. (2013) can predict the bond strength of confined sand-coated GFRP bars; however, *f*_*R*_ should be equal to 0.11.

## Conclusions and Recommendations

In this study, 16 GFRP-RC full-scale beam specimens were tested under four-point bending to evaluate the bond strength of sand-coated bars with and without the presence of confining stirrups. The lap-splice lengths used were equal to 40d_b_, 60d_b_, and 80d_b_; the stirrups were spaced on 100- and 200-mm centers. Based on the outcomes of this study, the following conclusions can be drawn:

- The reference GFRP-RC beams failed due to concrete crushing, whereas most of the spliced beams failed either by splitting of the concrete in the splice zone or rebar slippage.
- The presence of stirrups increased the bond strength, decreased longitudinal bar slippage, and increased splitting stresses. Specimens with splice length of 60 d_b_ and stirrups at 100 mm on centers showed 57% higher capacity than those with same splice length but without stirrups.
- The ACI 440.11 equation for bond strength overestimated the bond strength of the sand-coated GFRP bars and provided conservative results for beams with closely spaced stirrups. In the current experimental program, ACI 440.11 equation resulted in test-to-predicted ratio equal to 0.8 and standard deviation 0.17.
- The CSA S6:25 equation for bond strength predicted bond-strength values that were closer to the experimental results than the CSA S6:19 and CSA S806:12 equations. Similar to CSA S6:19, the bond-strength equation in CSA S6:25 takes into consideration the effect of confining stirrups. In this study, it was overshadowed by an upper limit on the combined contribution of the concrete cover and stirrups.
- The equation proposed by Esfahani et al. (2013) produced results consistent with the experimental results for the beams without stirrups. In contrast, it overestimated the capacity of the beams reinforced with sand-coated GFRP bars when GFRP stirrups were placed in the spliced region. Therefore, updates to the surface modification factor to include the effect of stirrup confinement are proposed. The average test-to-predicted ratio with updated modification factor was found to be equal to 1.1 and standard deviation 0.14.
- Based on the above outcomes of this study, the ACI 440.11 equation has conservatively ignored the effect of stirrups on the bond strength. Therefore, it is imperative to update the equation to include the effect of stirrups so as to avoid challenges in the implementation of design as per code.
- FRP-RC technology is growing at a fast pace, including various surface treatments and types. Investigating the bond for each bar type is difficult and gives rise to uncertainties. Currently, ASTM material specification provides minimum requirements for bond strength. This may not be sufficient, and it would be preferable to have standardized surface configurations as for the case of steel reinforcement. This would make the procurement process much simpler for the contractor.

## Data Availability

Data will be made available on request.
